# Bis(acetato-κ^2^ *O*,*O*′)diphen­yl(pyridine-κ*N*)tin(IV)

**DOI:** 10.1107/S1600536810007014

**Published:** 2010-03-03

**Authors:** Hamid Khaledi, Hapipah Mohd Ali, Mahmood A. Abdulla

**Affiliations:** a Department of Chemistry, University of Malaya, 50603 Kuala Lumpur, Malaysia; b Department of Molecular Medicine, University of Malaya, 50603 Kuala Lumpur, Malaysia

## Abstract

The asymmetric unit of the title compound, [Sn(C_6_H_5_)_2_(C_2_H_3_O_2_)_2_(C_5_H_5_N)], contains two crystallography independent mol­ecules. In both mol­ecules, the Sn^IV^ atom is seven-coordinated in a distorted penta­gonal-bipyramidal geometry with the two phenyl groups in axial positions. The two mol­ecules differ mainly in the torsion of the phenyl and pyridine rings. The dihredral angles between the phenyl rings are 89.54 (15) and 60.11 (14)° in the two mol­ecules while the dihedral angles between the pyridine rings and the acetate groups are 12.6 (2) and 41.77 (13)° in the two mol­ecules.

## Related literature

For the crystal structures of other diphenyl­tin(IV) complexes, see: Alcock *et al.* (1992 ▶); Gao *et al.* (2009 ▶); Li *et al.* (2009 ▶).
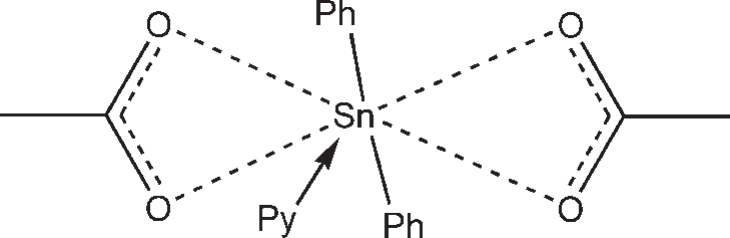


## Experimental

### 

#### Crystal data

[Sn(C_6_H_5_)_2_(C_2_H_3_O_2_)_2_(C_5_H_5_N)]
*M* *_r_* = 470.10
Monoclinic, 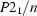

*a* = 9.7579 (1) Å
*b* = 32.7007 (5) Å
*c* = 12.9241 (2) Å
β = 91.650 (2)°
*V* = 4122.24 (10) Å^3^
*Z* = 8
Mo *K*α radiation
μ = 1.26 mm^−1^
*T* = 296 K
0.57 × 0.29 × 0.07 mm

#### Data collection

Bruker APEXII CCD diffractometer
Absorption correction: multi-scan (*SADABS*; Sheldrick, 1996 ▶) *T*_min_ = 0.533, *T*_max_ = 0.922
32617 measured reflections
7269 independent reflections
5389 reflections with *I* > 2σ(*I*)
*R*_int_ = 0.059

#### Refinement

*R*[*F* ^2^ > 2σ(*F* ^2^)] = 0.033
*wR*(*F* ^2^) = 0.067
*S* = 1.01
7269 reflections
491 parameters
H-atom parameters constrained
Δρ_max_ = 0.29 e Å^−3^
Δρ_min_ = −0.38 e Å^−3^

### 

Data collection: *APEX2* (Bruker, 2007 ▶); cell refinement: *SAINT* (Bruker, 2007 ▶); data reduction: *SAINT*; program(s) used to solve structure: *SHELXS97* (Sheldrick, 2008 ▶); program(s) used to refine structure: *SHELXL97* (Sheldrick, 2008 ▶); molecular graphics: *X-SEED* (Barbour, 2001 ▶); software used to prepare material for publication: *publCIF* (Westrip, 2010 ▶).

## Supplementary Material

Crystal structure: contains datablocks I, global. DOI: 10.1107/S1600536810007014/om2322sup1.cif

Structure factors: contains datablocks I. DOI: 10.1107/S1600536810007014/om2322Isup2.hkl

Additional supplementary materials:  crystallographic information; 3D view; checkCIF report

## Figures and Tables

**Fig. 1.**
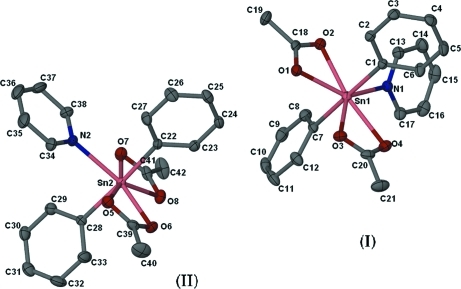
Thermal ellipsoid plot of the title compound at the 25% probability level. Hydrogen atoms have been omitted for clarity.

## supplementary crystallographic information

### Comment

In the crystal structure of the title compound two independent molecules with different conformations exist. In both molecules the Sn^IV^ atoms are hepta-coordinated by the four acetate O atoms, the two phenyl C atoms and the pyridine N atom, leading to an approximate pentagonal-bipyramidal configuration. The acetate and pyridine groups are positioned in the equatorial plane, while the phenyl groups occupy the axial positions. The two molecules differ mainly in torsion of the phenyl and pyridine rings. Thus, while the two phenyl groups in the Sn1 molecule are almost perpendicular with respect to each other, making a dihedral angle of 89.54 (15)°, the analogous angle in the Sn2 molecule is 60.11 (14)°. Moreover, in the Sn1 molecule, the pyridine ring is nearly coplanar with the plane passing through the acetate groups [dihedral angle = 12.6 (2)°], whereas in the Sn2 molecule they are highly twisted, the dihedral angle between the two planes being 41.77 (13)°.

### Experimental

The title compound was obtained as a colorless crystal by slow evaporation of a solution of diphenyltin(IV) dichloride in a mixture of pyridine and acetic acid at room temperature.

### Refinement

A low angle reflection, (-1 0 1), probably affected by extinction, was omitted from the dataset. Hydrogen atoms were placed at calculated positions (C–H 0.93–0.96 Å), and were treated as riding on their parent atoms, with U(H) set to 1.2–1.5 times Ueq(C).

### Crystal data

**Table:**

[Sn(C_6_H_5_)_2_(C_2_H_3_O_2_)_2_(C_5_H_5_N)]	*F*(000) = 1888
*M**_r_* = 470.10	*D*_x_ = 1.515 Mg m^−^^3^
Monoclinic, *P*2_1_/*n*	Mo *K*α radiation, λ = 0.71073 Å
Hall symbol: -P 2yn	Cell parameters from 5049 reflections
*a* = 9.7579 (1) Å	θ = 2.5–21.5°
*b* = 32.7007 (5) Å	µ = 1.26 mm^−^^1^
*c* = 12.9241 (2) Å	*T* = 296 K
β = 91.650 (2)°	Plate, colourless
*V* = 4122.24 (10) Å^3^	0.57 × 0.29 × 0.07 mm
*Z* = 8	

### Data collection

**Table:**

Bruker APEXII CCD diffractometer	7269 independent reflections
Radiation source: fine-focus sealed tube	5389 reflections with *I* > 2σ(*I*)
graphite	*R*_int_ = 0.059
φ and ω scans	θ_max_ = 25.0°, θ_min_ = 1.3°
Absorption correction: multi-scan (*SADABS*; Sheldrick, 1996)	*h* = −11→11
*T*_min_ = 0.533, *T*_max_ = 0.922	*k* = −38→38
32617 measured reflections	*l* = −15→15

### Refinement

**Table:**

Refinement on *F*^2^	Primary atom site location: structure-invariant direct methods
Least-squares matrix: full	Secondary atom site location: difference Fourier map
*R*[*F*^2^ > 2σ(*F*^2^)] = 0.033	Hydrogen site location: inferred from neighbouring sites
*wR*(*F*^2^) = 0.067	H-atom parameters constrained
*S* = 1.01	*w* = 1/[σ^2^(*F*_o_^2^) + (0.0384*P*)^2^] where *P* = (*F*_o_^2^ + 2*F*_c_^2^)/3
7269 reflections	(Δ/σ)_max_ = 0.002
491 parameters	Δρ_max_ = 0.29 e Å^−^^3^
0 restraints	Δρ_min_ = −0.38 e Å^−^^3^

### Special details

**Table:**

Geometry. All esds (except the esd in the dihedral angle between two l.s. planes) are estimated using the full covariance matrix. The cell esds are taken into account individually in the estimation of esds in distances, angles and torsion angles; correlations between esds in cell parameters are only used when they are defined by crystal symmetry. An approximate (isotropic) treatment of cell esds is used for estimating esds involving l.s. planes.
Refinement. Refinement of *F*^2^ against ALL reflections. The weighted *R*-factor wR and goodness of fit *S* are based on *F*^2^, conventional *R*-factors *R* are based on *F*, with *F* set to zero for negative *F*^2^. The threshold expression of *F*^2^ > σ(*F*^2^) is used only for calculating *R*-factors(gt) etc. and is not relevant to the choice of reflections for refinement. *R*-factors based on *F*^2^ are statistically about twice as large as those based on *F*, and *R*- factors based on ALL data will be even larger.

### Fractional atomic coordinates and isotropic or equivalent isotropic displacement parameters (Å^2^)

**Table:**

	*x*	*y*	*z*	*U*_iso_*/*U*_eq_	
Sn1	−0.08099 (3)	0.238659 (8)	0.55511 (2)	0.03786 (8)	
O1	0.1356 (3)	0.21752 (8)	0.5281 (2)	0.0490 (7)	
O2	0.1106 (3)	0.26740 (8)	0.6384 (2)	0.0506 (7)	
O3	−0.1368 (3)	0.19069 (8)	0.4356 (2)	0.0541 (7)	
O4	−0.3065 (3)	0.22409 (9)	0.5023 (2)	0.0549 (8)	
N1	−0.1946 (3)	0.28823 (10)	0.6576 (2)	0.0420 (8)	
C1	−0.0743 (4)	0.28432 (11)	0.4375 (3)	0.0361 (9)	
C2	0.0447 (4)	0.29306 (12)	0.3865 (3)	0.0429 (10)	
H2	0.1236	0.2781	0.4023	0.051*	
C3	0.0489 (4)	0.32350 (13)	0.3129 (3)	0.0507 (11)	
H3	0.1297	0.3286	0.2787	0.061*	
C4	−0.0656 (5)	0.34623 (13)	0.2901 (3)	0.0537 (11)	
H4	−0.0622	0.3674	0.2422	0.064*	
C5	−0.1860 (5)	0.33760 (14)	0.3386 (3)	0.0630 (13)	
H5	−0.2649	0.3524	0.3220	0.076*	
C6	−0.1896 (4)	0.30704 (13)	0.4118 (3)	0.0566 (12)	
H6	−0.2713	0.3016	0.4445	0.068*	
C7	−0.0925 (4)	0.19671 (12)	0.6810 (3)	0.0419 (10)	
C8	−0.0622 (4)	0.20869 (14)	0.7815 (3)	0.0567 (12)	
H8	−0.0408	0.2359	0.7953	0.068*	
C9	−0.0632 (4)	0.18058 (18)	0.8627 (4)	0.0670 (14)	
H9	−0.0447	0.1891	0.9303	0.080*	
C10	−0.0917 (5)	0.14038 (17)	0.8421 (4)	0.0723 (15)	
H10	−0.0897	0.1214	0.8957	0.087*	
C11	−0.1229 (5)	0.12801 (14)	0.7439 (4)	0.0751 (15)	
H11	−0.1441	0.1008	0.7305	0.090*	
C12	−0.1230 (5)	0.15597 (13)	0.6642 (3)	0.0582 (12)	
H12	−0.1442	0.1472	0.5972	0.070*	
C13	−0.1427 (4)	0.32547 (13)	0.6701 (3)	0.0530 (11)	
H13	−0.0601	0.3315	0.6393	0.064*	
C14	−0.2047 (5)	0.35533 (15)	0.7259 (4)	0.0701 (14)	
H14	−0.1663	0.3813	0.7318	0.084*	
C15	−0.3252 (6)	0.34605 (18)	0.7731 (4)	0.0755 (16)	
H15	−0.3695	0.3656	0.8123	0.091*	
C16	−0.3790 (5)	0.30764 (17)	0.7615 (4)	0.0688 (14)	
H16	−0.4602	0.3006	0.7930	0.083*	
C17	−0.3118 (4)	0.27970 (14)	0.7030 (3)	0.0528 (11)	
H17	−0.3493	0.2537	0.6946	0.063*	
C18	0.1870 (4)	0.24260 (13)	0.5926 (3)	0.0463 (10)	
C19	0.3381 (4)	0.24312 (15)	0.6149 (4)	0.0809 (16)	
H19A	0.3853	0.2345	0.5545	0.121*	
H19B	0.3597	0.2249	0.6712	0.121*	
H19C	0.3664	0.2703	0.6333	0.121*	
C20	−0.2642 (5)	0.19806 (14)	0.4401 (3)	0.0535 (12)	
C21	−0.3630 (5)	0.17626 (16)	0.3673 (4)	0.0805 (16)	
H21A	−0.4542	0.1785	0.3927	0.121*	
H21B	−0.3379	0.1479	0.3629	0.121*	
H21C	−0.3598	0.1885	0.2999	0.121*	
Sn2	0.30045 (2)	0.002342 (8)	0.745640 (19)	0.03527 (8)	
O5	0.3221 (3)	−0.03640 (8)	0.5930 (2)	0.0504 (7)	
O6	0.1207 (3)	−0.01786 (9)	0.6446 (2)	0.0498 (7)	
O7	0.3408 (3)	0.03830 (8)	0.8982 (2)	0.0550 (8)	
O8	0.1310 (3)	0.02980 (9)	0.8407 (2)	0.0535 (7)	
N2	0.5450 (3)	−0.00126 (10)	0.7521 (3)	0.0450 (8)	
C22	0.3157 (4)	0.05580 (11)	0.6515 (3)	0.0364 (9)	
C23	0.2074 (4)	0.08331 (12)	0.6459 (3)	0.0486 (11)	
H23	0.1305	0.0787	0.6851	0.058*	
C24	0.2118 (4)	0.11739 (12)	0.5835 (3)	0.0533 (11)	
H24	0.1387	0.1356	0.5814	0.064*	
C25	0.3232 (5)	0.12439 (13)	0.5249 (3)	0.0559 (12)	
H25	0.3266	0.1476	0.4834	0.067*	
C26	0.4303 (5)	0.09725 (14)	0.5269 (3)	0.0621 (13)	
H26	0.5057	0.1019	0.4861	0.075*	
C27	0.4263 (4)	0.06299 (13)	0.5897 (3)	0.0517 (11)	
H27	0.4990	0.0446	0.5904	0.062*	
C28	0.2980 (4)	−0.05365 (11)	0.8314 (3)	0.0397 (9)	
C29	0.3981 (4)	−0.06247 (13)	0.9064 (3)	0.0539 (11)	
H29	0.4661	−0.0433	0.9218	0.065*	
C30	0.3979 (5)	−0.09943 (15)	0.9587 (3)	0.0608 (13)	
H30	0.4662	−0.1050	1.0084	0.073*	
C31	0.2995 (5)	−0.12750 (15)	0.9381 (4)	0.0661 (13)	
H31	0.3004	−0.1523	0.9732	0.079*	
C32	0.1997 (6)	−0.11938 (15)	0.8666 (4)	0.0788 (16)	
H32	0.1312	−0.1386	0.8531	0.095*	
C33	0.1988 (5)	−0.08257 (14)	0.8130 (4)	0.0681 (14)	
H33	0.1295	−0.0775	0.7638	0.082*	
C34	0.6104 (4)	−0.02641 (13)	0.6902 (4)	0.0591 (12)	
H34	0.5591	−0.0433	0.6461	0.071*	
C35	0.7505 (5)	−0.02857 (17)	0.6884 (5)	0.0839 (17)	
H35	0.7932	−0.0461	0.6427	0.101*	
C36	0.8260 (5)	−0.00474 (19)	0.7544 (5)	0.092 (2)	
H36	0.9213	−0.0061	0.7552	0.111*	
C37	0.7620 (5)	0.02105 (18)	0.8191 (5)	0.0818 (17)	
H37	0.8120	0.0375	0.8650	0.098*	
C38	0.6208 (4)	0.02225 (14)	0.8152 (4)	0.0613 (12)	
H38	0.5765	0.0403	0.8586	0.074*	
C39	0.1937 (5)	−0.03631 (12)	0.5796 (3)	0.0486 (11)	
C40	0.1267 (6)	−0.05751 (16)	0.4898 (4)	0.0949 (19)	
H40A	0.1922	−0.0613	0.4366	0.142*	
H40B	0.0517	−0.0412	0.4634	0.142*	
H40C	0.0931	−0.0836	0.5115	0.142*	
C41	0.2146 (5)	0.04391 (13)	0.9085 (3)	0.0541 (12)	
C42	0.1632 (6)	0.06754 (16)	0.9989 (3)	0.0914 (19)	
H42A	0.0770	0.0564	1.0192	0.137*	
H42B	0.1512	0.0957	0.9797	0.137*	
H42C	0.2285	0.0656	1.0557	0.137*	

### Atomic displacement parameters (Å^2^)

**Table:**

	*U*^11^	*U*^22^	*U*^33^	*U*^12^	*U*^13^	*U*^23^
Sn1	0.03559 (15)	0.03860 (16)	0.03957 (16)	−0.00013 (12)	0.00445 (11)	0.00538 (12)
O1	0.0409 (16)	0.0492 (18)	0.0568 (19)	0.0040 (14)	0.0035 (14)	0.0052 (14)
O2	0.0454 (17)	0.0497 (18)	0.0567 (19)	0.0005 (14)	−0.0009 (14)	0.0027 (14)
O3	0.0514 (19)	0.0558 (19)	0.0555 (19)	−0.0115 (15)	0.0057 (15)	−0.0031 (14)
O4	0.0430 (17)	0.065 (2)	0.056 (2)	−0.0068 (15)	0.0027 (14)	0.0017 (16)
N1	0.044 (2)	0.042 (2)	0.040 (2)	0.0020 (16)	0.0037 (16)	0.0024 (15)
C1	0.037 (2)	0.040 (2)	0.032 (2)	0.0012 (18)	−0.0010 (17)	0.0015 (16)
C2	0.036 (2)	0.048 (3)	0.044 (3)	0.0013 (19)	0.0022 (19)	0.0003 (19)
C3	0.047 (3)	0.059 (3)	0.046 (3)	−0.009 (2)	0.002 (2)	0.011 (2)
C4	0.070 (3)	0.048 (3)	0.042 (3)	−0.005 (2)	−0.003 (2)	0.016 (2)
C5	0.058 (3)	0.068 (3)	0.063 (3)	0.021 (2)	0.005 (2)	0.023 (3)
C6	0.046 (3)	0.067 (3)	0.057 (3)	0.014 (2)	0.015 (2)	0.020 (2)
C7	0.040 (2)	0.041 (3)	0.045 (3)	0.0058 (19)	0.0096 (19)	0.0047 (19)
C8	0.051 (3)	0.065 (3)	0.054 (3)	−0.004 (2)	−0.001 (2)	0.011 (2)
C9	0.054 (3)	0.099 (4)	0.047 (3)	0.006 (3)	−0.003 (2)	0.019 (3)
C10	0.068 (3)	0.076 (4)	0.075 (4)	0.026 (3)	0.027 (3)	0.040 (3)
C11	0.098 (4)	0.044 (3)	0.086 (4)	0.018 (3)	0.037 (3)	0.021 (3)
C12	0.078 (3)	0.043 (3)	0.054 (3)	0.010 (2)	0.022 (2)	0.000 (2)
C13	0.053 (3)	0.051 (3)	0.056 (3)	0.004 (2)	0.002 (2)	0.000 (2)
C14	0.078 (4)	0.052 (3)	0.079 (4)	0.013 (3)	−0.005 (3)	−0.014 (3)
C15	0.080 (4)	0.090 (4)	0.056 (3)	0.036 (3)	−0.003 (3)	−0.024 (3)
C16	0.060 (3)	0.095 (4)	0.052 (3)	0.013 (3)	0.007 (2)	−0.006 (3)
C17	0.049 (3)	0.061 (3)	0.049 (3)	0.004 (2)	0.012 (2)	0.008 (2)
C18	0.038 (2)	0.048 (3)	0.053 (3)	−0.001 (2)	−0.003 (2)	0.025 (2)
C19	0.040 (3)	0.088 (4)	0.114 (4)	0.006 (3)	−0.013 (3)	0.022 (3)
C20	0.058 (3)	0.056 (3)	0.046 (3)	−0.025 (2)	−0.003 (2)	0.011 (2)
C21	0.081 (4)	0.098 (4)	0.062 (3)	−0.040 (3)	−0.015 (3)	−0.001 (3)
Sn2	0.03129 (14)	0.03723 (16)	0.03719 (15)	0.00157 (12)	−0.00107 (11)	−0.00432 (12)
O5	0.0536 (19)	0.0474 (18)	0.0504 (18)	0.0029 (14)	0.0036 (14)	−0.0118 (13)
O6	0.0387 (16)	0.0607 (19)	0.0496 (18)	−0.0053 (14)	−0.0063 (14)	−0.0022 (15)
O7	0.057 (2)	0.057 (2)	0.0508 (18)	0.0061 (15)	−0.0069 (15)	−0.0132 (14)
O8	0.0469 (18)	0.067 (2)	0.0473 (18)	0.0136 (15)	0.0049 (14)	−0.0012 (15)
N2	0.0338 (18)	0.044 (2)	0.057 (2)	0.0029 (16)	−0.0019 (16)	0.0013 (17)
C22	0.037 (2)	0.035 (2)	0.037 (2)	0.0041 (17)	−0.0048 (18)	−0.0057 (17)
C23	0.042 (2)	0.048 (3)	0.055 (3)	−0.001 (2)	−0.005 (2)	−0.003 (2)
C24	0.053 (3)	0.040 (3)	0.066 (3)	0.009 (2)	−0.010 (2)	0.004 (2)
C25	0.080 (3)	0.042 (3)	0.046 (3)	0.006 (2)	−0.005 (2)	0.006 (2)
C26	0.076 (3)	0.059 (3)	0.053 (3)	0.005 (3)	0.025 (2)	0.005 (2)
C27	0.056 (3)	0.048 (3)	0.052 (3)	0.017 (2)	0.014 (2)	0.004 (2)
C28	0.044 (2)	0.037 (2)	0.039 (2)	0.0005 (19)	0.0048 (19)	0.0002 (17)
C29	0.055 (3)	0.054 (3)	0.052 (3)	−0.002 (2)	−0.005 (2)	0.004 (2)
C30	0.062 (3)	0.066 (3)	0.054 (3)	0.013 (3)	−0.002 (2)	0.015 (2)
C31	0.084 (4)	0.055 (3)	0.060 (3)	0.006 (3)	0.010 (3)	0.015 (2)
C32	0.097 (4)	0.056 (3)	0.082 (4)	−0.032 (3)	−0.012 (3)	0.017 (3)
C33	0.067 (3)	0.067 (3)	0.070 (3)	−0.018 (3)	−0.021 (3)	0.017 (3)
C34	0.043 (3)	0.057 (3)	0.079 (3)	0.011 (2)	0.007 (2)	−0.002 (2)
C35	0.046 (3)	0.091 (4)	0.115 (5)	0.020 (3)	0.017 (3)	0.016 (4)
C36	0.037 (3)	0.116 (6)	0.124 (6)	0.003 (3)	0.006 (3)	0.046 (4)
C37	0.047 (3)	0.090 (4)	0.107 (5)	−0.027 (3)	−0.019 (3)	0.026 (4)
C38	0.047 (3)	0.063 (3)	0.074 (3)	−0.009 (2)	−0.003 (2)	0.000 (3)
C39	0.062 (3)	0.039 (3)	0.043 (3)	−0.009 (2)	−0.009 (2)	0.0013 (19)
C40	0.129 (5)	0.091 (4)	0.063 (4)	−0.030 (4)	−0.028 (3)	−0.024 (3)
C41	0.072 (3)	0.048 (3)	0.042 (3)	0.018 (2)	0.002 (2)	0.004 (2)
C42	0.138 (5)	0.092 (4)	0.044 (3)	0.050 (4)	0.009 (3)	−0.015 (3)

### Geometric parameters (Å, °)

**Table:**

Sn1—C1	2.133 (4)	Sn2—C22	2.138 (4)
Sn1—C7	2.134 (4)	Sn2—C28	2.141 (4)
Sn1—O3	2.257 (3)	Sn2—O6	2.255 (2)
Sn1—O1	2.260 (3)	Sn2—O8	2.273 (3)
Sn1—O2	2.328 (3)	Sn2—O7	2.320 (3)
Sn1—O4	2.334 (3)	Sn2—O5	2.359 (2)
Sn1—N1	2.387 (3)	Sn2—N2	2.388 (3)
Sn1—C18	2.649 (4)	Sn2—C41	2.661 (4)
Sn1—C20	2.649 (4)	O5—C39	1.260 (5)
O1—C18	1.263 (5)	O6—C39	1.270 (5)
O2—C18	1.261 (5)	O7—C41	1.256 (5)
O3—C20	1.269 (5)	O8—C41	1.267 (5)
O4—C20	1.250 (5)	N2—C34	1.324 (5)
N1—C13	1.327 (5)	N2—C38	1.330 (5)
N1—C17	1.330 (5)	C22—C27	1.381 (5)
C1—C6	1.381 (5)	C22—C23	1.388 (5)
C1—C2	1.381 (5)	C23—C24	1.377 (5)
C2—C3	1.379 (5)	C23—H23	0.9300
C2—H2	0.9300	C24—C25	1.362 (6)
C3—C4	1.367 (5)	C24—H24	0.9300
C3—H3	0.9300	C25—C26	1.371 (6)
C4—C5	1.377 (6)	C25—H25	0.9300
C4—H4	0.9300	C26—C27	1.385 (5)
C5—C6	1.377 (5)	C26—H26	0.9300
C5—H5	0.9300	C27—H27	0.9300
C6—H6	0.9300	C28—C33	1.369 (5)
C7—C8	1.380 (5)	C28—C29	1.387 (5)
C7—C12	1.381 (5)	C29—C30	1.385 (6)
C8—C9	1.395 (6)	C29—H29	0.9300
C8—H8	0.9300	C30—C31	1.349 (6)
C9—C10	1.368 (7)	C30—H30	0.9300
C9—H9	0.9300	C31—C32	1.350 (6)
C10—C11	1.358 (7)	C31—H31	0.9300
C10—H10	0.9300	C32—C33	1.389 (6)
C11—C12	1.378 (6)	C32—H32	0.9300
C11—H11	0.9300	C33—H33	0.9300
C12—H12	0.9300	C34—C35	1.370 (6)
C13—C14	1.365 (6)	C34—H34	0.9300
C13—H13	0.9300	C35—C36	1.357 (7)
C14—C15	1.374 (7)	C35—H35	0.9300
C14—H14	0.9300	C36—C37	1.353 (8)
C15—C16	1.368 (7)	C36—H36	0.9300
C15—H15	0.9300	C37—C38	1.378 (6)
C16—C17	1.366 (6)	C37—H37	0.9300
C16—H16	0.9300	C38—H38	0.9300
C17—H17	0.9300	C39—C40	1.486 (5)
C18—C19	1.494 (5)	C40—H40A	0.9600
C19—H19A	0.9600	C40—H40B	0.9600
C19—H19B	0.9600	C40—H40C	0.9600
C19—H19C	0.9600	C41—C42	1.500 (6)
C20—C21	1.507 (5)	C42—H42A	0.9600
C21—H21A	0.9600	C42—H42B	0.9600
C21—H21B	0.9600	C42—H42C	0.9600
C21—H21C	0.9600		
			
C1—Sn1—C7	175.45 (14)	C20—C21—H21B	109.5
C1—Sn1—O3	90.59 (12)	H21A—C21—H21B	109.5
C7—Sn1—O3	93.34 (13)	C20—C21—H21C	109.5
C1—Sn1—O1	93.22 (12)	H21A—C21—H21C	109.5
C7—Sn1—O1	89.49 (12)	H21B—C21—H21C	109.5
O3—Sn1—O1	83.69 (11)	C22—Sn2—C28	175.00 (14)
C1—Sn1—O2	90.37 (11)	C22—Sn2—O6	88.61 (12)
C7—Sn1—O2	88.06 (13)	C28—Sn2—O6	91.65 (12)
O3—Sn1—O2	140.50 (10)	C22—Sn2—O8	92.76 (12)
O1—Sn1—O2	56.84 (10)	C28—Sn2—O8	92.23 (12)
C1—Sn1—O4	88.99 (12)	O6—Sn2—O8	82.10 (11)
C7—Sn1—O4	91.25 (13)	C22—Sn2—O7	93.23 (12)
O3—Sn1—O4	56.72 (10)	C28—Sn2—O7	89.88 (12)
O1—Sn1—O4	140.38 (11)	O6—Sn2—O7	138.65 (10)
O2—Sn1—O4	162.77 (10)	O8—Sn2—O7	56.55 (10)
C1—Sn1—N1	86.82 (12)	C22—Sn2—O5	87.36 (11)
C7—Sn1—N1	88.72 (13)	C28—Sn2—O5	88.62 (12)
O3—Sn1—N1	138.17 (11)	O6—Sn2—O5	56.34 (10)
O1—Sn1—N1	138.13 (11)	O8—Sn2—O5	138.43 (10)
O2—Sn1—N1	81.30 (10)	O7—Sn2—O5	164.99 (10)
O4—Sn1—N1	81.48 (11)	C22—Sn2—N2	88.55 (12)
C1—Sn1—C18	92.64 (13)	C28—Sn2—N2	88.04 (13)
C7—Sn1—C18	88.00 (13)	O6—Sn2—N2	139.96 (11)
O3—Sn1—C18	112.11 (13)	O8—Sn2—N2	137.93 (11)
O1—Sn1—C18	28.43 (11)	O7—Sn2—N2	81.39 (11)
O2—Sn1—C18	28.42 (11)	O5—Sn2—N2	83.64 (11)
O4—Sn1—C18	168.76 (13)	C22—Sn2—C41	93.49 (13)
N1—Sn1—C18	109.71 (13)	C28—Sn2—C41	91.10 (13)
C1—Sn1—C20	89.13 (13)	O6—Sn2—C41	110.48 (13)
C7—Sn1—C20	93.23 (14)	O8—Sn2—C41	28.39 (11)
O3—Sn1—C20	28.58 (12)	O7—Sn2—C41	28.17 (11)
O1—Sn1—C20	112.27 (14)	O5—Sn2—C41	166.79 (13)
O2—Sn1—C20	169.05 (14)	N2—Sn2—C41	109.55 (14)
O4—Sn1—C20	28.15 (12)	C39—O5—Sn2	90.1 (2)
N1—Sn1—C20	109.59 (14)	C39—O6—Sn2	94.6 (2)
C18—Sn1—C20	140.70 (16)	C41—O7—Sn2	91.2 (2)
C18—O1—Sn1	93.2 (2)	C41—O8—Sn2	93.0 (2)
C18—O2—Sn1	90.1 (2)	C34—N2—C38	117.4 (4)
C20—O3—Sn1	93.1 (3)	C34—N2—Sn2	120.5 (3)
C20—O4—Sn1	90.1 (3)	C38—N2—Sn2	122.0 (3)
C13—N1—C17	118.0 (4)	C27—C22—C23	117.8 (4)
C13—N1—Sn1	120.6 (3)	C27—C22—Sn2	122.4 (3)
C17—N1—Sn1	121.4 (3)	C23—C22—Sn2	119.6 (3)
C6—C1—C2	117.7 (4)	C24—C23—C22	121.2 (4)
C6—C1—Sn1	120.4 (3)	C24—C23—H23	119.4
C2—C1—Sn1	121.9 (3)	C22—C23—H23	119.4
C3—C2—C1	121.4 (4)	C25—C24—C23	120.1 (4)
C3—C2—H2	119.3	C25—C24—H24	120.0
C1—C2—H2	119.3	C23—C24—H24	120.0
C4—C3—C2	120.0 (4)	C24—C25—C26	120.0 (4)
C4—C3—H3	120.0	C24—C25—H25	120.0
C2—C3—H3	120.0	C26—C25—H25	120.0
C3—C4—C5	119.6 (4)	C25—C26—C27	120.1 (4)
C3—C4—H4	120.2	C25—C26—H26	120.0
C5—C4—H4	120.2	C27—C26—H26	120.0
C6—C5—C4	120.0 (4)	C22—C27—C26	120.8 (4)
C6—C5—H5	120.0	C22—C27—H27	119.6
C4—C5—H5	120.0	C26—C27—H27	119.6
C5—C6—C1	121.2 (4)	C33—C28—C29	117.3 (4)
C5—C6—H6	119.4	C33—C28—Sn2	121.3 (3)
C1—C6—H6	119.4	C29—C28—Sn2	121.4 (3)
C8—C7—C12	117.5 (4)	C30—C29—C28	120.8 (4)
C8—C7—Sn1	121.4 (3)	C30—C29—H29	119.6
C12—C7—Sn1	121.0 (3)	C28—C29—H29	119.6
C7—C8—C9	121.0 (5)	C31—C30—C29	120.6 (4)
C7—C8—H8	119.5	C31—C30—H30	119.7
C9—C8—H8	119.5	C29—C30—H30	119.7
C10—C9—C8	119.5 (5)	C32—C31—C30	119.7 (4)
C10—C9—H9	120.2	C32—C31—H31	120.1
C8—C9—H9	120.2	C30—C31—H31	120.1
C11—C10—C9	120.4 (4)	C31—C32—C33	120.5 (5)
C11—C10—H10	119.8	C31—C32—H32	119.8
C9—C10—H10	119.8	C33—C32—H32	119.8
C10—C11—C12	119.8 (5)	C28—C33—C32	121.2 (4)
C10—C11—H11	120.1	C28—C33—H33	119.4
C12—C11—H11	120.1	C32—C33—H33	119.4
C11—C12—C7	121.8 (5)	N2—C34—C35	122.8 (5)
C11—C12—H12	119.1	N2—C34—H34	118.6
C7—C12—H12	119.1	C35—C34—H34	118.6
N1—C13—C14	123.2 (4)	C36—C35—C34	118.9 (5)
N1—C13—H13	118.4	C36—C35—H35	120.5
C14—C13—H13	118.4	C34—C35—H35	120.5
C13—C14—C15	118.3 (5)	C37—C36—C35	119.6 (5)
C13—C14—H14	120.8	C37—C36—H36	120.2
C15—C14—H14	120.8	C35—C36—H36	120.2
C16—C15—C14	119.0 (5)	C36—C37—C38	118.3 (5)
C16—C15—H15	120.5	C36—C37—H37	120.8
C14—C15—H15	120.5	C38—C37—H37	120.8
C17—C16—C15	119.1 (5)	N2—C38—C37	122.9 (5)
C17—C16—H16	120.5	N2—C38—H38	118.5
C15—C16—H16	120.5	C37—C38—H38	118.5
N1—C17—C16	122.5 (5)	O5—C39—O6	119.0 (4)
N1—C17—H17	118.8	O5—C39—C40	121.3 (4)
C16—C17—H17	118.8	O6—C39—C40	119.7 (4)
O2—C18—O1	119.9 (4)	C39—C40—H40A	109.5
O2—C18—C19	119.7 (4)	C39—C40—H40B	109.5
O1—C18—C19	120.4 (4)	H40A—C40—H40B	109.5
O2—C18—Sn1	61.5 (2)	C39—C40—H40C	109.5
O1—C18—Sn1	58.4 (2)	H40A—C40—H40C	109.5
C19—C18—Sn1	177.8 (3)	H40B—C40—H40C	109.5
C18—C19—H19A	109.5	O7—C41—O8	119.2 (4)
C18—C19—H19B	109.5	O7—C41—C42	120.6 (4)
H19A—C19—H19B	109.5	O8—C41—C42	120.2 (4)
C18—C19—H19C	109.5	O7—C41—Sn2	60.7 (2)
H19A—C19—H19C	109.5	O8—C41—Sn2	58.6 (2)
H19B—C19—H19C	109.5	C42—C41—Sn2	178.7 (4)
O4—C20—O3	120.1 (4)	C41—C42—H42A	109.5
O4—C20—C21	120.5 (5)	C41—C42—H42B	109.5
O3—C20—C21	119.4 (5)	H42A—C42—H42B	109.5
O4—C20—Sn1	61.8 (2)	C41—C42—H42C	109.5
O3—C20—Sn1	58.3 (2)	H42A—C42—H42C	109.5
C21—C20—Sn1	175.5 (3)	H42B—C42—H42C	109.5
C20—C21—H21A	109.5		
			
C1—Sn1—O1—C18	89.6 (2)	C18—Sn1—C20—O4	177.4 (2)
C7—Sn1—O1—C18	−86.8 (2)	C1—Sn1—C20—O3	92.8 (2)
O3—Sn1—O1—C18	179.8 (2)	C7—Sn1—C20—O3	−91.1 (2)
O2—Sn1—O1—C18	1.3 (2)	O1—Sn1—C20—O3	−0.3 (3)
O4—Sn1—O1—C18	−178.1 (2)	O2—Sn1—C20—O3	5.4 (7)
N1—Sn1—O1—C18	0.8 (3)	O4—Sn1—C20—O3	−177.7 (4)
C20—Sn1—O1—C18	180.0 (2)	N1—Sn1—C20—O3	179.1 (2)
C1—Sn1—O2—C18	−94.9 (2)	C18—Sn1—C20—O3	−0.3 (3)
C7—Sn1—O2—C18	89.4 (2)	C1—Sn1—C20—C21	32 (5)
O3—Sn1—O2—C18	−3.5 (3)	C7—Sn1—C20—C21	−152 (5)
O1—Sn1—O2—C18	−1.3 (2)	O3—Sn1—C20—C21	−61 (5)
O4—Sn1—O2—C18	177.4 (3)	O1—Sn1—C20—C21	−61 (5)
N1—Sn1—O2—C18	178.4 (2)	O2—Sn1—C20—C21	−56 (5)
C20—Sn1—O2—C18	−7.6 (7)	O4—Sn1—C20—C21	121 (5)
C1—Sn1—O3—C20	−87.1 (2)	N1—Sn1—C20—C21	118 (5)
C7—Sn1—O3—C20	90.6 (3)	C18—Sn1—C20—C21	−61 (5)
O1—Sn1—O3—C20	179.7 (2)	C22—Sn2—O5—C39	−90.3 (2)
O2—Sn1—O3—C20	−178.4 (2)	C28—Sn2—O5—C39	92.7 (2)
O4—Sn1—O3—C20	1.3 (2)	O6—Sn2—O5—C39	−0.2 (2)
N1—Sn1—O3—C20	−1.2 (3)	O8—Sn2—O5—C39	0.9 (3)
C18—Sn1—O3—C20	179.8 (2)	O7—Sn2—O5—C39	177.1 (3)
C1—Sn1—O4—C20	90.0 (3)	N2—Sn2—O5—C39	−179.1 (2)
C7—Sn1—O4—C20	−94.5 (3)	C41—Sn2—O5—C39	3.8 (6)
O3—Sn1—O4—C20	−1.3 (2)	C22—Sn2—O6—C39	87.9 (2)
O1—Sn1—O4—C20	−3.8 (3)	C28—Sn2—O6—C39	−87.1 (2)
O2—Sn1—O4—C20	178.0 (3)	O8—Sn2—O6—C39	−179.1 (2)
N1—Sn1—O4—C20	177.0 (3)	O7—Sn2—O6—C39	−178.8 (2)
C18—Sn1—O4—C20	−8.4 (7)	O5—Sn2—O6—C39	0.2 (2)
C1—Sn1—N1—C13	−50.5 (3)	N2—Sn2—O6—C39	1.9 (3)
C7—Sn1—N1—C13	128.6 (3)	C41—Sn2—O6—C39	−178.8 (2)
O3—Sn1—N1—C13	−137.8 (3)	C22—Sn2—O7—C41	91.4 (3)
O1—Sn1—N1—C13	40.8 (4)	C28—Sn2—O7—C41	−92.6 (3)
O2—Sn1—N1—C13	40.4 (3)	O6—Sn2—O7—C41	−0.2 (3)
O4—Sn1—N1—C13	−139.9 (3)	O8—Sn2—O7—C41	0.2 (2)
C18—Sn1—N1—C13	41.2 (3)	O5—Sn2—O7—C41	−176.8 (3)
C20—Sn1—N1—C13	−138.4 (3)	N2—Sn2—O7—C41	179.4 (3)
C1—Sn1—N1—C17	128.8 (3)	C22—Sn2—O8—C41	−92.2 (3)
C7—Sn1—N1—C17	−52.1 (3)	C28—Sn2—O8—C41	88.2 (3)
O3—Sn1—N1—C17	41.5 (4)	O6—Sn2—O8—C41	179.5 (3)
O1—Sn1—N1—C17	−139.9 (3)	O7—Sn2—O8—C41	−0.2 (2)
O2—Sn1—N1—C17	−140.3 (3)	O5—Sn2—O8—C41	178.6 (2)
O4—Sn1—N1—C17	39.4 (3)	N2—Sn2—O8—C41	−1.4 (3)
C18—Sn1—N1—C17	−139.5 (3)	C22—Sn2—N2—C34	−99.0 (3)
C20—Sn1—N1—C17	40.9 (3)	C28—Sn2—N2—C34	77.4 (3)
C7—Sn1—C1—C6	−58.1 (19)	O6—Sn2—N2—C34	−12.9 (4)
O3—Sn1—C1—C6	91.7 (3)	O8—Sn2—N2—C34	168.5 (3)
O1—Sn1—C1—C6	175.4 (3)	O7—Sn2—N2—C34	167.5 (3)
O2—Sn1—C1—C6	−127.8 (3)	O5—Sn2—N2—C34	−11.5 (3)
O4—Sn1—C1—C6	35.0 (3)	C41—Sn2—N2—C34	167.8 (3)
N1—Sn1—C1—C6	−46.5 (3)	C22—Sn2—N2—C38	79.3 (3)
C18—Sn1—C1—C6	−156.1 (3)	C28—Sn2—N2—C38	−104.3 (3)
C20—Sn1—C1—C6	63.1 (3)	O6—Sn2—N2—C38	165.4 (3)
C7—Sn1—C1—C2	120.1 (17)	O8—Sn2—N2—C38	−13.2 (4)
O3—Sn1—C1—C2	−90.1 (3)	O7—Sn2—N2—C38	−14.2 (3)
O1—Sn1—C1—C2	−6.4 (3)	O5—Sn2—N2—C38	166.8 (3)
O2—Sn1—C1—C2	50.4 (3)	C41—Sn2—N2—C38	−13.9 (3)
O4—Sn1—C1—C2	−146.8 (3)	C28—Sn2—C22—C27	−20.5 (18)
N1—Sn1—C1—C2	131.7 (3)	O6—Sn2—C22—C27	−113.5 (3)
C18—Sn1—C1—C2	22.0 (3)	O8—Sn2—C22—C27	164.5 (3)
C20—Sn1—C1—C2	−118.7 (3)	O7—Sn2—C22—C27	107.8 (3)
C6—C1—C2—C3	0.3 (6)	O5—Sn2—C22—C27	−57.1 (3)
Sn1—C1—C2—C3	−178.0 (3)	N2—Sn2—C22—C27	26.6 (3)
C1—C2—C3—C4	1.1 (6)	C41—Sn2—C22—C27	136.1 (3)
C2—C3—C4—C5	−2.2 (7)	C28—Sn2—C22—C23	155.2 (15)
C3—C4—C5—C6	1.9 (7)	O6—Sn2—C22—C23	62.1 (3)
C4—C5—C6—C1	−0.5 (7)	O8—Sn2—C22—C23	−19.9 (3)
C2—C1—C6—C5	−0.6 (6)	O7—Sn2—C22—C23	−76.5 (3)
Sn1—C1—C6—C5	177.7 (3)	O5—Sn2—C22—C23	118.5 (3)
C1—Sn1—C7—C8	−32 (2)	N2—Sn2—C22—C23	−157.8 (3)
O3—Sn1—C7—C8	177.8 (3)	C41—Sn2—C22—C23	−48.3 (3)
O1—Sn1—C7—C8	94.1 (3)	C27—C22—C23—C24	−2.1 (6)
O2—Sn1—C7—C8	37.3 (3)	Sn2—C22—C23—C24	−177.9 (3)
O4—Sn1—C7—C8	−125.5 (3)	C22—C23—C24—C25	0.7 (6)
N1—Sn1—C7—C8	−44.1 (3)	C23—C24—C25—C26	0.8 (7)
C18—Sn1—C7—C8	65.7 (3)	C24—C25—C26—C27	−1.0 (7)
C20—Sn1—C7—C8	−153.6 (3)	C23—C22—C27—C26	1.9 (6)
C1—Sn1—C7—C12	151.2 (17)	Sn2—C22—C27—C26	177.6 (3)
O3—Sn1—C7—C12	1.5 (3)	C25—C26—C27—C22	−0.5 (7)
O1—Sn1—C7—C12	−82.2 (3)	C22—Sn2—C28—C33	−96.9 (16)
O2—Sn1—C7—C12	−139.0 (3)	O6—Sn2—C28—C33	−4.0 (4)
O4—Sn1—C7—C12	58.2 (3)	O8—Sn2—C28—C33	78.1 (4)
N1—Sn1—C7—C12	139.6 (3)	O7—Sn2—C28—C33	134.7 (4)
C18—Sn1—C7—C12	−110.6 (3)	O5—Sn2—C28—C33	−60.3 (4)
C20—Sn1—C7—C12	30.1 (3)	N2—Sn2—C28—C33	−144.0 (4)
C12—C7—C8—C9	−0.3 (6)	C41—Sn2—C28—C33	106.5 (4)
Sn1—C7—C8—C9	−176.7 (3)	C22—Sn2—C28—C29	81.9 (16)
C7—C8—C9—C10	1.4 (7)	O6—Sn2—C28—C29	174.8 (3)
C8—C9—C10—C11	−2.0 (7)	O8—Sn2—C28—C29	−103.1 (3)
C9—C10—C11—C12	1.4 (8)	O7—Sn2—C28—C29	−46.5 (3)
C10—C11—C12—C7	−0.2 (7)	O5—Sn2—C28—C29	118.5 (3)
C8—C7—C12—C11	−0.3 (6)	N2—Sn2—C28—C29	34.8 (3)
Sn1—C7—C12—C11	176.1 (3)	C41—Sn2—C28—C29	−74.7 (3)
C17—N1—C13—C14	−0.8 (6)	C33—C28—C29—C30	1.2 (6)
Sn1—N1—C13—C14	178.5 (3)	Sn2—C28—C29—C30	−177.7 (3)
N1—C13—C14—C15	1.3 (7)	C28—C29—C30—C31	−0.5 (7)
C13—C14—C15—C16	−0.7 (7)	C29—C30—C31—C32	−0.5 (7)
C14—C15—C16—C17	−0.3 (7)	C30—C31—C32—C33	0.9 (8)
C13—N1—C17—C16	−0.3 (6)	C29—C28—C33—C32	−0.8 (7)
Sn1—N1—C17—C16	−179.6 (3)	Sn2—C28—C33—C32	178.1 (4)
C15—C16—C17—N1	0.8 (7)	C31—C32—C33—C28	−0.2 (8)
Sn1—O2—C18—O1	2.2 (4)	C38—N2—C34—C35	−0.7 (7)
Sn1—O2—C18—C19	−177.9 (3)	Sn2—N2—C34—C35	177.7 (4)
Sn1—O1—C18—O2	−2.2 (4)	N2—C34—C35—C36	1.7 (8)
Sn1—O1—C18—C19	177.8 (3)	C34—C35—C36—C37	−1.1 (9)
C1—Sn1—C18—O2	85.9 (2)	C35—C36—C37—C38	−0.2 (8)
C7—Sn1—C18—O2	−89.6 (2)	C34—N2—C38—C37	−0.7 (7)
O3—Sn1—C18—O2	177.6 (2)	Sn2—N2—C38—C37	−179.1 (3)
O1—Sn1—C18—O2	177.8 (4)	C36—C37—C38—N2	1.2 (8)
O4—Sn1—C18—O2	−176.0 (5)	Sn2—O5—C39—O6	0.3 (4)
N1—Sn1—C18—O2	−1.7 (2)	Sn2—O5—C39—C40	−179.3 (4)
C20—Sn1—C18—O2	177.7 (2)	Sn2—O6—C39—O5	−0.3 (4)
C1—Sn1—C18—O1	−91.9 (2)	Sn2—O6—C39—C40	179.3 (4)
C7—Sn1—C18—O1	92.6 (2)	Sn2—O7—C41—O8	−0.3 (4)
O3—Sn1—C18—O1	−0.2 (2)	Sn2—O7—C41—C42	−179.4 (4)
O2—Sn1—C18—O1	−177.8 (4)	Sn2—O8—C41—O7	0.3 (4)
O4—Sn1—C18—O1	6.2 (7)	Sn2—O8—C41—C42	179.5 (4)
N1—Sn1—C18—O1	−179.5 (2)	C22—Sn2—C41—O7	−90.3 (3)
C20—Sn1—C18—O1	0.0 (3)	C28—Sn2—C41—O7	87.7 (3)
C1—Sn1—C18—C19	−150 (10)	O6—Sn2—C41—O7	179.9 (2)
C7—Sn1—C18—C19	34 (10)	O8—Sn2—C41—O7	−179.7 (4)
O3—Sn1—C18—C19	−58 (10)	O5—Sn2—C41—O7	176.3 (4)
O1—Sn1—C18—C19	−58 (9)	N2—Sn2—C41—O7	−0.6 (3)
O2—Sn1—C18—C19	124 (10)	C22—Sn2—C41—O8	89.3 (2)
O4—Sn1—C18—C19	−52 (10)	C28—Sn2—C41—O8	−92.7 (3)
N1—Sn1—C18—C19	122 (10)	O6—Sn2—C41—O8	−0.5 (3)
C20—Sn1—C18—C19	−58 (10)	O7—Sn2—C41—O8	179.7 (4)
Sn1—O4—C20—O3	2.3 (4)	O5—Sn2—C41—O8	−4.0 (6)
Sn1—O4—C20—C21	−175.5 (4)	N2—Sn2—C41—O8	179.0 (2)
Sn1—O3—C20—O4	−2.4 (4)	C22—Sn2—C41—C42	68 (16)
Sn1—O3—C20—C21	175.5 (3)	C28—Sn2—C41—C42	−114 (16)
C1—Sn1—C20—O4	−89.5 (3)	O6—Sn2—C41—C42	−22 (16)
C7—Sn1—C20—O4	86.6 (3)	O8—Sn2—C41—C42	−21 (16)
O3—Sn1—C20—O4	177.7 (4)	O7—Sn2—C41—C42	159 (16)
O1—Sn1—C20—O4	177.4 (2)	O5—Sn2—C41—C42	−25 (17)
O2—Sn1—C20—O4	−176.9 (5)	N2—Sn2—C41—C42	158 (16)
N1—Sn1—C20—O4	−3.2 (3)		

## References

[bb1] Alcock, N. W., Culver, J. & Roe, S. M. (1992). *J. Chem. Soc. Dalton Trans.* pp. 1477–1484.

[bb2] Barbour, L. J. (2001). *J. Supramol. Chem* **1**, 189–191.

[bb3] Bruker (2007). *APEX2* and *SAINT* Bruker AXS Inc., Madison, Wisconsin, USA.

[bb4] Gao, Z., Zhai, X., Zhou, F. & Cheng, Z. (2009). *Acta Cryst.* E**65**, m1134. 10.1107/S1600536809033170 PMC2969951 21577471

[bb5] Li, J., Yin, H., Wen, L. & Cui, J. (2009). *Acta Cryst.* E**65**, m1441. 10.1107/S1600536809043591 PMC2971108 21578172

[bb6] Sheldrick, G. M. (1996). *SADABS* University of Göttingen, Germany.

[bb7] Sheldrick, G. M. (2008). *Acta Cryst.* A**64**, 112–122. 10.1107/S0108767307043930 18156677

[bb8] Westrip, S. P. (2010). *publCIF* In preparation.

